# SwinOCSR: end-to-end optical chemical structure recognition using a Swin Transformer

**DOI:** 10.1186/s13321-022-00624-5

**Published:** 2022-07-01

**Authors:** Zhanpeng Xu, Jianhua Li, Zhaopeng Yang, Shiliang Li, Honglin Li

**Affiliations:** 1 School of Information Science and Engineering, East China University of Science and Technology, 130 Mei Long Road, Shanghai, 200237 China; 2 State Key Laboratory of Bioreactor Engineering, Shanghai Key Laboratory of New Drug Design, School of Pharmacy, East China University of Science and Technology, Shanghai, 200237 China

**Keywords:** Chemical Structure Recognition, Deep Learning, Swin Transfromer, End-to-End Model

## Abstract

Optical chemical structure recognition from scientific publications is essential for rediscovering a chemical structure. It is an extremely challenging problem, and current rule-based and deep-learning methods cannot achieve satisfactory recognition rates. Herein, we propose SwinOCSR, an end-to-end model based on a Swin Transformer. This model uses the Swin Transformer as the backbone to extract image features and introduces Transformer models to convert chemical information from publications into DeepSMILES. A novel chemical structure dataset was constructed to train and verify our method. Our proposed Swin Transformer-based model was extensively tested against the backbone of existing publicly available deep learning methods. The experimental results show that our model significantly outperforms the compared methods, demonstrating the model’s effectiveness. Moreover, we used a focal loss to address the token imbalance problem in the text representation of the chemical structure diagram, and our model achieved an accuracy of 98.58%.

## Introduction

Optical chemical structure recognition (OCSR) is the conversion of the chemical structure information of chemical compounds from scientific publications into machine-readable formats. Chemical structures printed in scientific publications are usually in image formats such as JPEG, PNG, and GIF. They cannot be directly utilized because they are not a machine-readable representation of molecules. The purpose of OCSR is to correctly translate this chemical structure information into a machine-readable representation and store them in a chemical information database. OCSR is time-consuming and error-prone, and requires domain knowledge to eliminate ambiguities in structures. As chemical structure scientific publications continue to increase exponentially, OCSR plays a vital role in many chemical subfields, such as synthetic science, natural product research, drug discovery and etc. Therefore, OCSR is still in high demand.

Existing automatic OCSR software systems include Kekule [1], OROCS [2], CLIDE [3], MLOCSR [4], ChemReader [5] and OSRA [6]. Most of these systems usually use rule-based approaches to recognize molecular diagrams.

With the rapid development of deep learning, both computer vision and natural language processing have become popular research topics in recent years. As a specific subdomain of computer vision, image captioning is used to identify the objects of an image firstly and then expresses the relationship among them in the form of accurate syntactically generated sentences. Image captioning often adopts a special framework, *i*.*e*., an encoder-decoder, of which the encoder usually uses a convolutional neural network (CNN), and the decoder usually use a Recurrent Neural Network (RNN). For example, in a neural image capture generator [7], a CNN-based InceptionNet [8] is used to extract image features, and an RNN is used to decode image features for text generation. The RNN can also be replaced with Long Short-Term Memory (LSTM) [9] or Gated Recurrent Unit (GRU) [10]. To improve the interpretability of neural networks, an attention mechanism is applied to the image captioning task [11]. The basic idea is to use convolutional layers to acquire image features and then weight them with attentions before sending them to an RNN for decoding. Using a self-attention mechanism, the Transformer [12] model proposed by Google has recently achieved an excellent performance in various translation-related tasks and has been widely applied.

Similar to image captioning, the OCSR task can be abstracted as the process of translating a chemical structure diagram into a computer-readable textual representation. Compared with image captioning, two major challenges of an OCSR task are complex chemical patterns in chemical structures and long corresponding chemical representation. The existing methods based on deep learning [13–16] use CNNs as their backbones to extract image features of molecules. However, CNN only learns local representation and cannot effectively use global information. To learn the global representation and obtain more comprehensive chemical structure information, we use Swin Transformer [17] as a backbone to extract image features of molecules. Moreover, one noticeable phenomenon is the frequency imbalance of elements in molecules. For example, C, H, and O appear more frequently, and Br, Cl, and Ar appear less frequently. This results in an imbalance of tokens in the text representation. We use a focal loss [18] to solve the imbalance problem. The contribution of this work can be summarized in three parts:

1. We present a new deep-learning OCSR (SwinOCSR) approach using a Swin Transformer as a backbone to extract image features and Transformer [12] to generate DeepSMILES [19] that are more syntactically valid than SMILES [20] in an end-to-end manner. Our method learns the global representation and obtain more abundant image features compared with other methods. It provides strong support for the subsequent Transformer part.
2. Based on the analysis of molecules, we use a focal loss to address the token imbalance problem in the text representation of molecular diagrams. This is the first attempt at explicitly optimizing such a problem in OCSR tasks, to the best of our knowledge.
3. We construct a novel chemical structure molecule dataset containing four categories (Kekule, Aromatic, substituent and Kekule, substituent and Aromatic), and our approach is robust against different molecule categories in constructed dataset. And, it is excellent in recognizing long-character chemical structures. The model trained on the constructed dataset achieved an accuracy of 98.58%.

## Related work

### Rule-based OCSR approaches

Early OCSR tasks used a rule-based approach. They used image processing techniques and optical character recognition for atomic labeling and charge recognition, encoded various rules for bond detection, and compiled connection tables. Kekule [1] was the first complete OCSR tool for scanning, vectorization, dashed and wedge line searches, optical character recognition, graphical editing, and post-processing. In addition, OSRA [6] is an open-source chemical structure extraction tool developed by the NCI. The extracted chemical structure can be directly converted into the SMILES or SDF format. Although OSRA can recognize some common group abbreviations, dashed lines, and wedge bonds, it cannot recognize charges or isotopes. CLIDE, ChemReader, CLiDE Pro [21], ChemInfty [22], and the approach by Sadawi et al. [23] made further improvements. However, these approaches have certain drawbacks. For example, the rule-based system will become difficult to interpret when molecular diagrams contain ambiguous or uncommon representations. As one of the current challenges, the various recognition components of a rule-based system are interdependent, making further improvements extremely difficult to achieve.

### Deep-learning-based OCSR approaches

Unlike rule-based approaches, deep learning-based methods identify chemical structures without hardcoded rules. For this reason, several deep learning-based OCSR methods have been proposed. In 2019, Staker et al*.* [13] presented the first deep learning-based OCSR approach, with a SMILES file as the output. Its accuracies on the validation sets ranged from 41% to 83%. However, this approach is closed-source and unavailable for re-testing, and it is only used to recognize low-resolution images. Img2Mol [24] is another deep learning-based OCSR approach, whose performance was verified by comparing it with the rule-based approaches. However, there is no comparison with exsiting deep learning-based methods, and it is preliminary. DECIMER [14] and subsequent DECIMER 1.0 [15] are two other deep-learning-based approaches. Based on existing show-and-tell deep neural networks, DECIMER uses Inception V3 [25] as a backbone to extract image features and GRU to predict SMILES. However, its performance does not yet rival the performance of existing traditional approaches. The updated version of DECIMER, DECIMER 1.0 [15], substitutes Inception V3 with EfficientNet-B3 [26] and GRU with Transformer. DECIMER 1.0 achieved a Tanimoto level of about 96% in a dataset of 30–35 million molecules. The latest deep-learning-based OCSR approach, Image2SMILES [16], uses ResNet-50 [27] as a backbone to extract image features and Transformer as a decoder part to predict FG-SMILES in the dataset of 10 million molecules. Image2SMILES achieved an accuracy of about 90.7%, but it still needs further improvement.

Among these approaches, many CNNs and their variants are used as backbones to extract image features in OCSR tasks. Therefore, a robust backbone is important for the OCSR task. The Swin Transformer model, a state-of-the-art backbone, surpasses the previous models in image classification, object detection and semantic segmentation. Hence, the Swin Transformer is chosen as our model backbone for OCSR.

### SwinOCSR: Deep-learning-based chemical structure diagram recognition approach

**Fig. 1:**
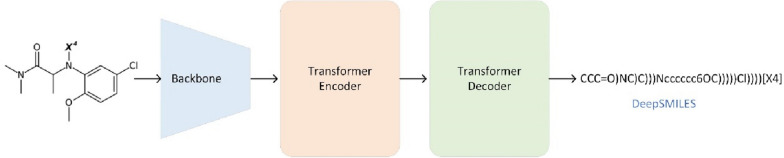
SwinOCSR framework for chemical structure recognition

The framework of our SwinOCSR approach for chemical structure diagram recognition is shown in Fig. 1. It consists of a backbone, Transformer encoder, and Transformer decoder. First, the backbone extracts image features from an input molecule image to obtain a high-dimensional patch sequence. Next, the patch sequence and positional embedding are fed into the Transformer encoder to output a representation sequence. Finally, the Transformer decoder uses the representation sequence to decode the corresponding DeepSMILES.

### Backbone

**Fig. 2:**
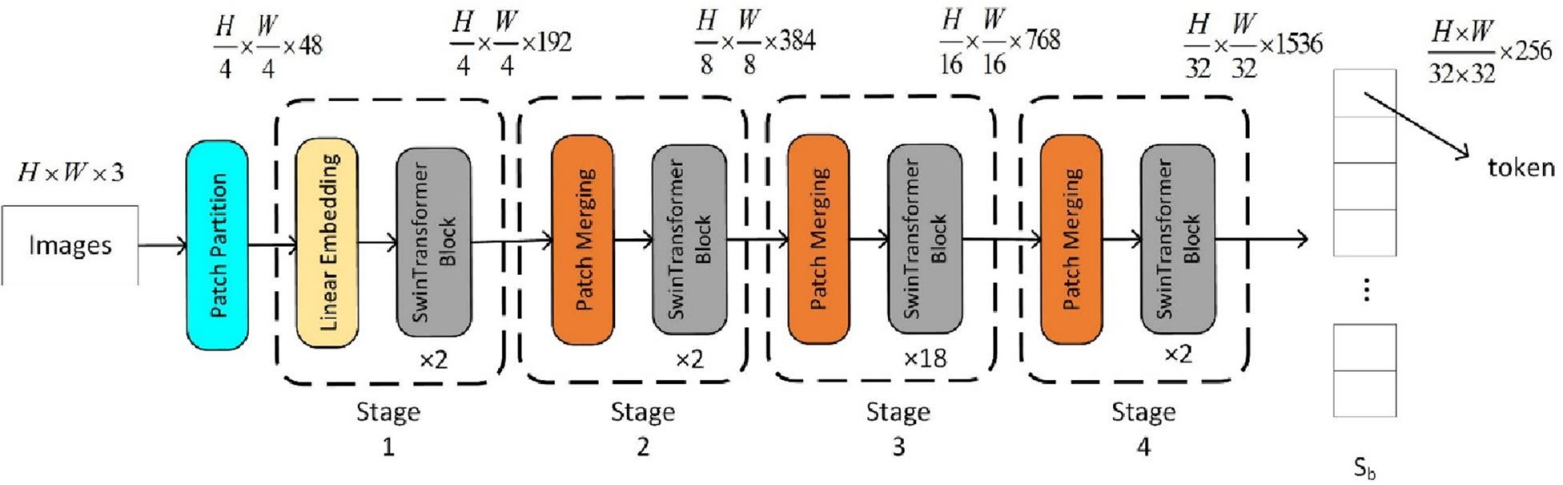
Swin Transformer-based backbone module

**Fig. 3:**
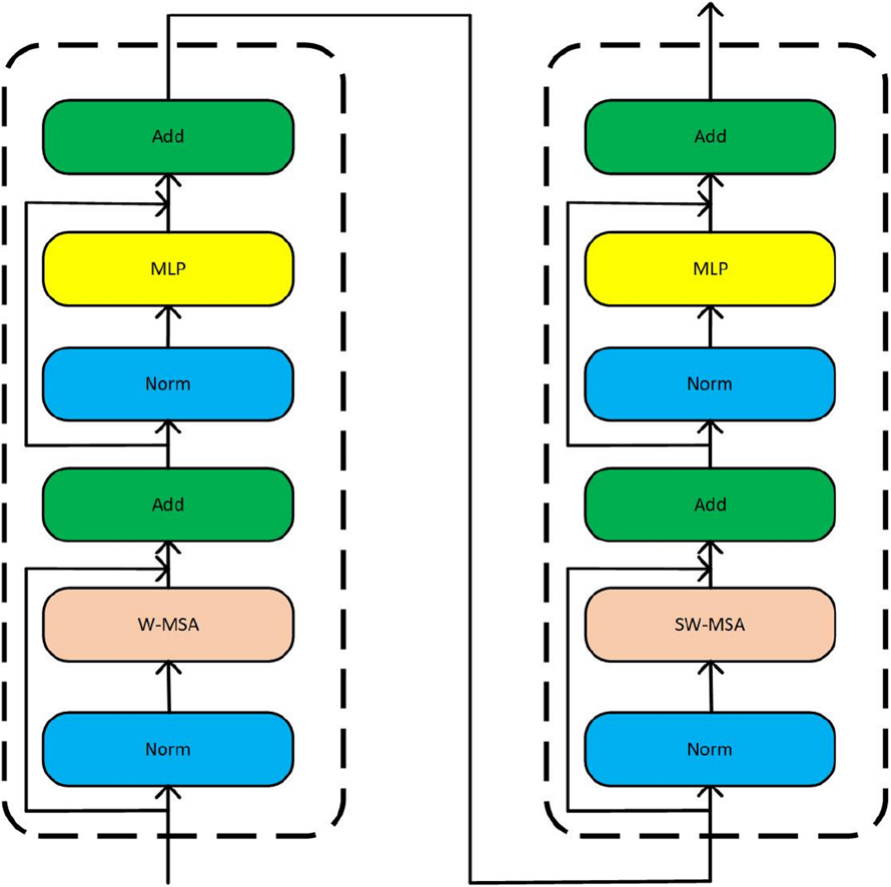
A Swin Transformer Block

The backbone is built based on the Swin Transformer, shown in Fig. 2. First, the molecule image is partitioned into non-overlapping patches, and the size of a patch is 4 × 4. Each patch is treated as a “token” and its feature is set as a concatenation of the raw pixel RGB value. After partitioning, a linear embedding layer is used to project this raw-valued feature to a certain dimension (192 for SwinOCSR), and several Swin Transformer blocks are used to extract feature information. As shown in Fig. 3, each Swin Transformer block contains two important modules, window multi-head self attention (W-MSA) and shift window multi-head self attention (SW-MSA) modules. W-MSA is used to extract local feature information in a window. SW-MSA is used to extract global feature information between windows. This means that Swin Transformer uses both local and global information, which greatly enhances Swin Transformer's feature extraction capabilities. This process is called “Stage 1.” In the following three stages, to generate hierarchical representation, Swin Transfromer does not use pooling which is usually used in CNN and may introduces information loss. Instead, it adopts merging neighboring patches to reduce the size of feature maps to avoid information loss. Finally, to construct a sequence as the encoder input, the feature in spatial dimensions is flattened, resulting in a sequence **S**_**b**_ that represents chemical structure information.

### Encoder

**Fig. 4:**
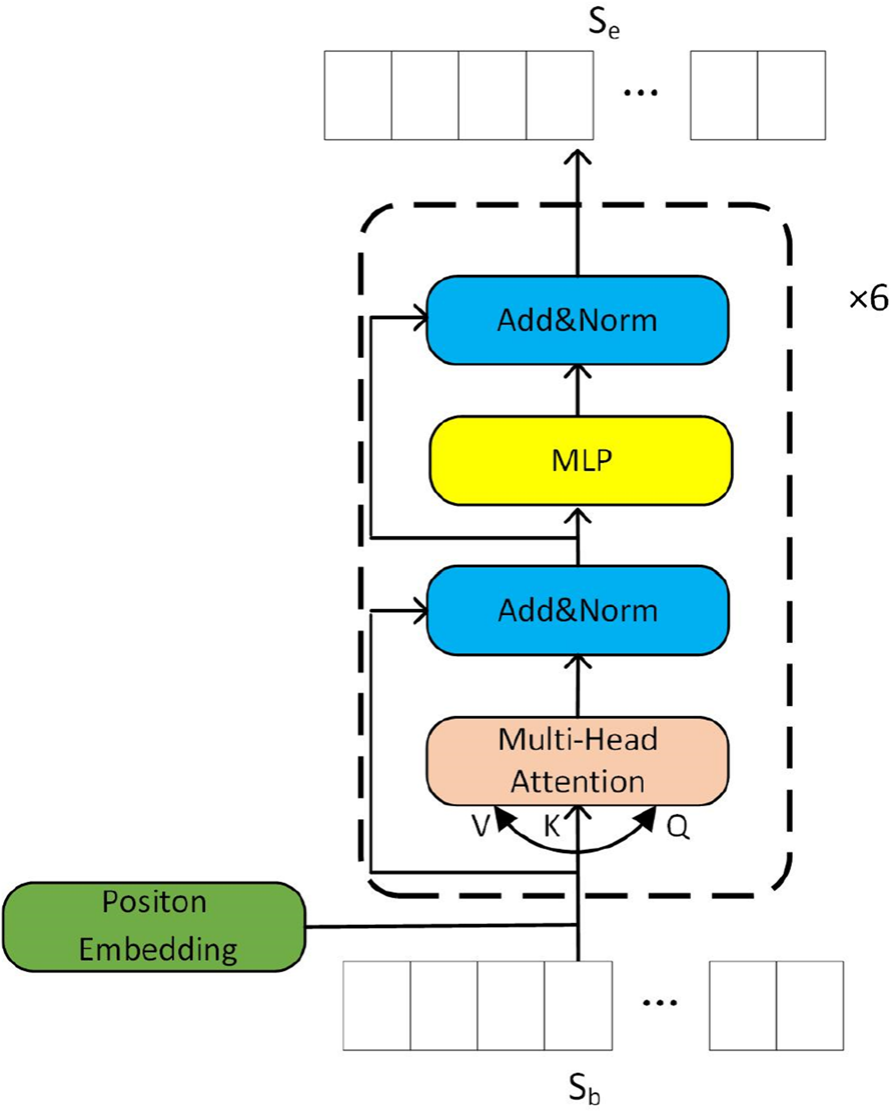
Transformer-based chemical structure encoder module

The encoding module consists of a positional encoding operation and six standard Transformer encoder layers. The six standard layers are linked sequentially, each of which contains two specific sublayers. The first sublayer is a multi-head attention layer, and the second is an MLP layer. Each sublayer is followed by a residual connection operation and a normalized operation, as shown in Fig. 4. The output **S**_**b**_ of the backbone flows into the first Transformer encoder sublayer. The Q, K, and V of the attention layer are obtained by multiplying the respective weight matrices of the three with **S**_**b**_. The attention function is then used to map the Q and a set of K-V pairs to an output. After obtaining the calculation results, the data are transferred to the MLP layer. The output sequence **S**_**e**_ of the encoder is obtained once all six standard layers are finished.

### Decoder

**Fig. 5:**
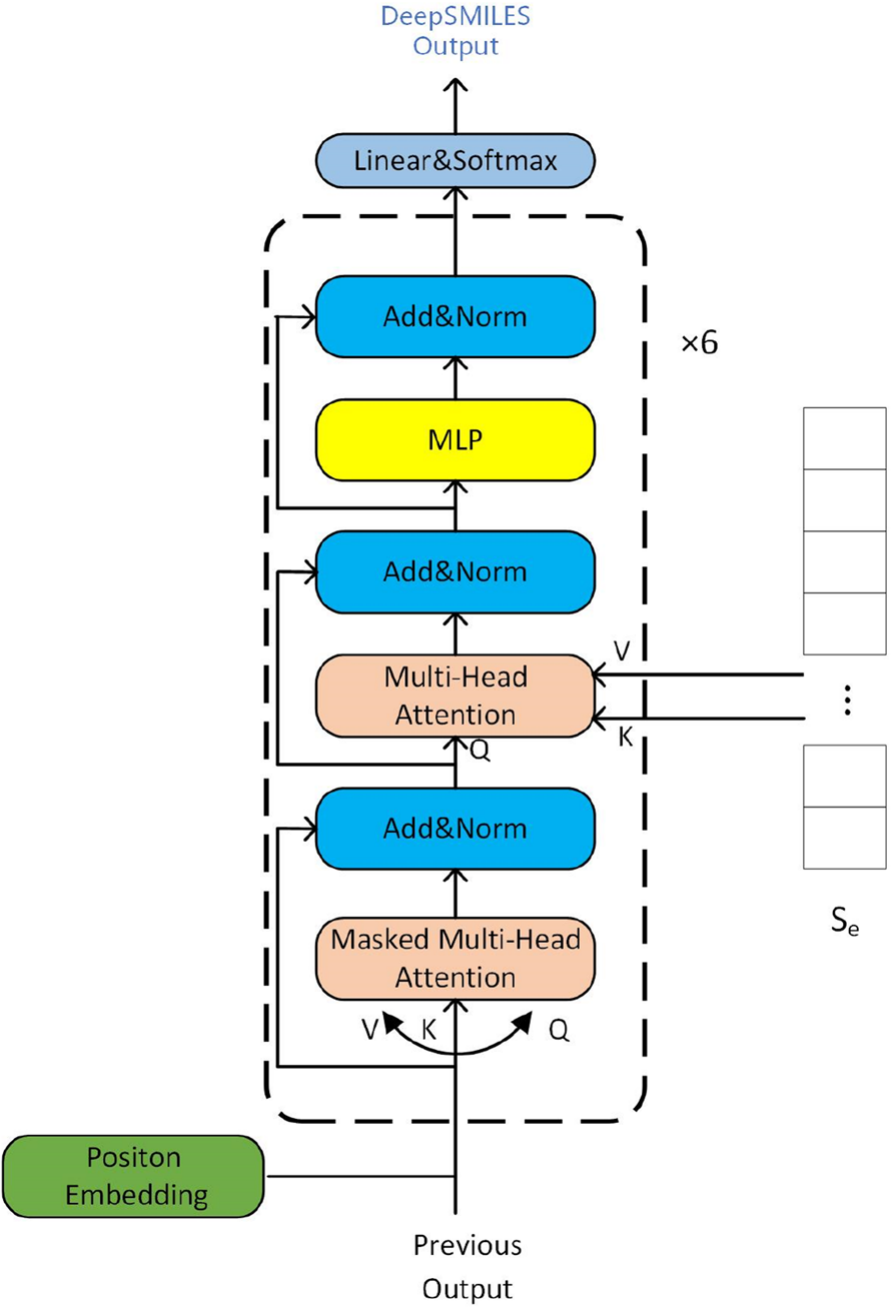
Transformer-based chemical structure decoder module

The decoding module consists of a positional encoding operation, a stack of six standard Transformer decoder layers, a linear layer and a softmax layer. A standard transformer decoder layer contains three specific sublayers. The first sublayer is a multi-head attention layer containing a mask. This mask ensures that the prediction of the position ***i*** depends only on the known outputs before the position ***i***. The second sublayer is a multi-head attention layer, and the third is an MLP layer. Similar to the encoding module, residual connections and layer normalization follow each sublayer, as shown in Fig. 5. Each time step in the decoding phase outputs a new token of the output sequence. At each time step, the previously generated output sequence (token sequence) flows into the first sublayer of Transformer decoder to learn internal relationships. The output of the first sublayer and the **S**_**e**_ sequence from the encoder are fed into the second sublayer of Transformer decoder to capture their relationships. Then, the result is transferred to the MLP layer. The output of Transformer decoder is obtained once all six standard layers are finished. And the output is fed into a linear layer and a softmax layer to obtain a token as the final output for this time step.

### Dataset

As described in the literature, the manual labeling of data is tedious, and it is difficult to obtain large numbers of data. Therefore, we did not directly extract molecular diagrams from studies on patents and other chemical publications to generate training data, but instead used cheminformatics toolkit CDK [28] to generate molecular diagrams.

**Fig. 6:**
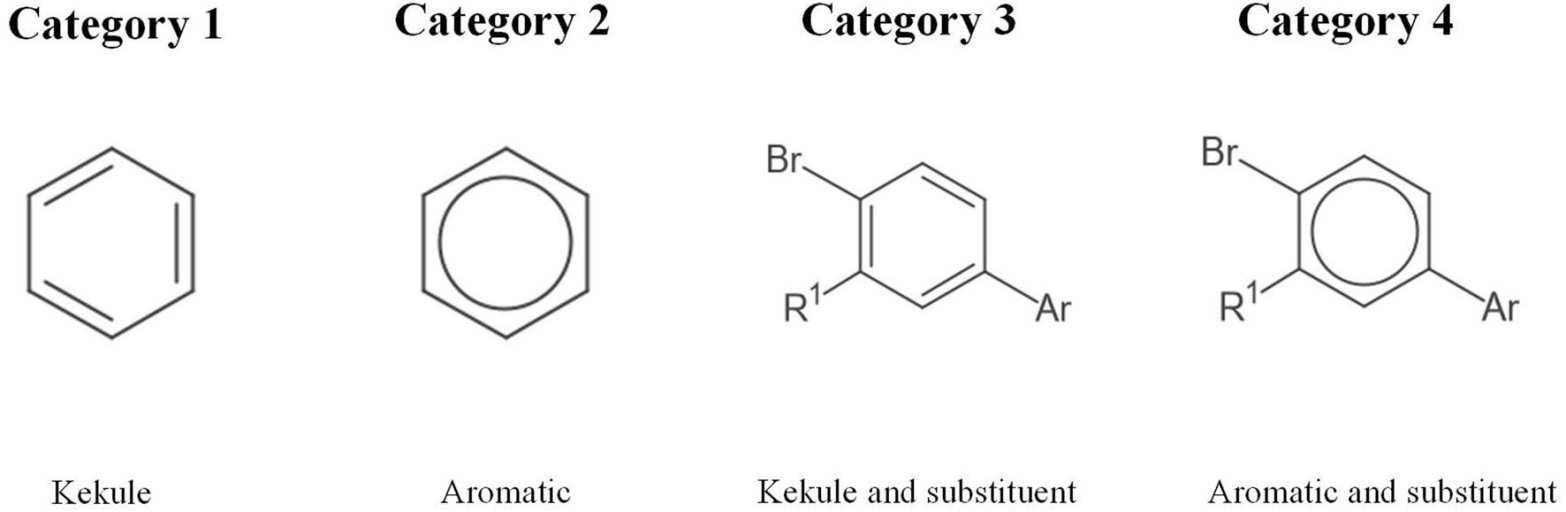
An example of each category of molecule data

An ideal generated dataset be as diverse as possible and should be similar to real molecular representation in publications. So, we used millions of molecules and both two different ring structures (Aromatic and Kekule) to make molecular diagrams diverse, and introduced substituents, which are widely used in patents, to generate molecular diagrams similar to those in patents. We have downloaded the first 8.5 million PubChem [29] structures (1–8,500,000 PubChem Indices) and gained 6,987,630 unique SMILES strings. Based on these SMILES files, we constructed a dataset of 5 million molecules and it consists of four categories of molecule data, each containing 1.25 million molecules. Table 1 shows the four categories of molecule data distinguished according to two criteria. One is whether most molecules include substituent; the other is that the ring structure is Aromatic or Kekule. Figure 6 shows an example of each category of molecule data.

**Table 1:** Description of each category of molecule data

Category Index	Substituent	Aromatic/Kekule	Size
1	×	Kekule	1,250,000
2	×	Aromatic	1,250,000
3	√	Kekule	1,250,000
4	√	Aromatic	1,250,000

The dataset was generated as follows: first, molecule SMILES files were downloaded from PubChem [29]. This kind of SMILES only contains Kekule ring and does not contain substituents, belonging to Category 1. Then, half of the downloaded SMILES files are canonically converted into SMILES strings including aromatic ring by RDKit, and these molecules in this kind of SMILES belong to Category 2. Next, some SMILES strings in Category 1 were broken, and some of both 224 substituents in the patent literature and common atom(s) with brackets ([Pb], [NH], [Ru], [Li], [K], [Si], [S +], [O], [O-], [N +], [N], [P], [C], [H], [2H], [3H], [B]) were randomly added to the broken SMILES strings, forming new SMILES strings which belong to Category 3. Category 4 is generated from Category 2 in the same way. Finally, for each category, molecules were converted into a canonical SMILES string, and 1,250,000 molecules with unique canonical SMILES were randomly chosen. The molecular canonical SMILES were then converted into DeepSMILES and used to render images using CDK.

**Fig. 7:**
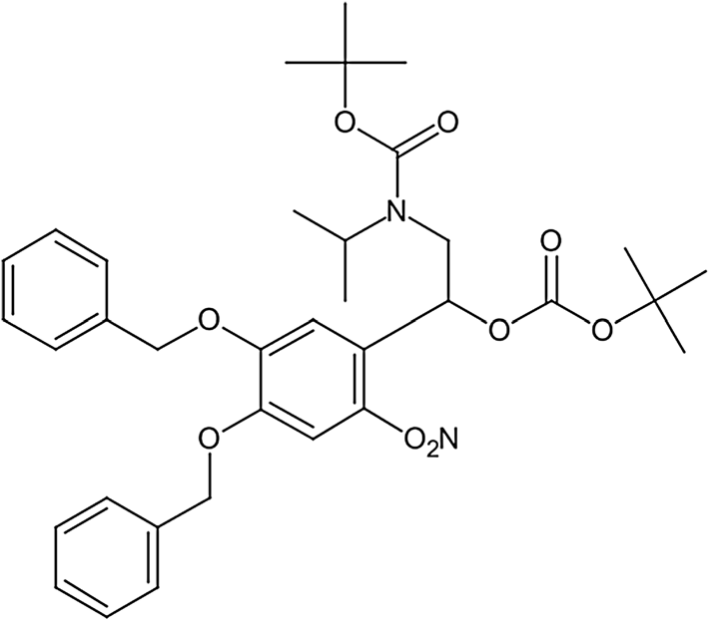
An example of generating a molecule diagram from CCC)NCCC = CC = CC = C6)OCC = CC = CC = C6)))))))))OCC = CC = CC = C6))))))))))[N +] = O)[O-]))))OC = O)OCC)C)C)))))))C = O)OCC)C)C

**Fig. 8:**
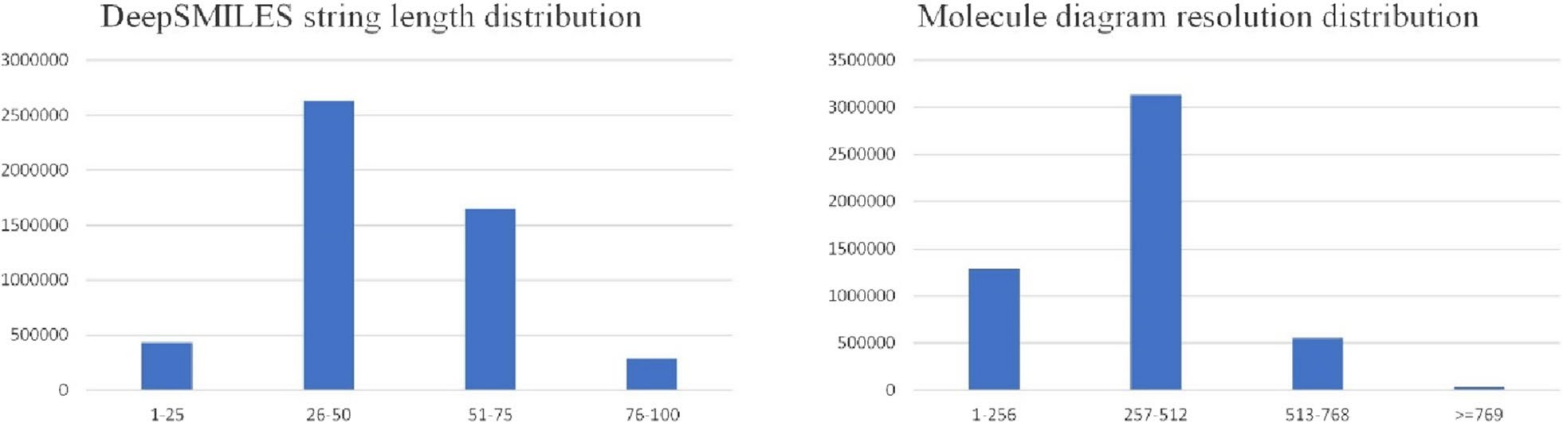
The distribution of the lengths of the DeepSMILES strings and resolution of molecular diagram

During image generation, some parameters (such as substituent fonts, subscripts, corner spacing, and size) of CDK image generation are set to make the molecule images closer to the images in the literature. In addition, specific rules for the condensed formula of the molecular structure are added to generate the chemical structure images. The generated chemical diagram is a four-channel diagram by default. Because the molecule diagram is black and white like a binary diagram, recognition of molecular diagram only requires its contour, without additional color channels. According to a threshold, all diagrams are changed into a binary diagram. To meet the input requirements of the model with three channels, we copied the one-channel binary diagram to pad each channel of three-channel diagram. Figure 7 shows an example of a generation molecular diagram. The length distribution of the DeepSMILES string and resolution of the molecular diagram in our dataset are shown in Fig. 8.

To evaluate our model, each category of the processed dataset is randomly spilt  in a ratio of 18:1:1 for training, validation and test, respectively, as shown in Table 2. The total size of the training set is 4500000, while both the size of the validation set and that of the test set are 250000. We selected four metrics per image for quantitative performance evaluation on accuracy, Tanimoto, BLEU [30], and ROUGE [31]. The first two metrics are two frequently used metrics for OCSR, and the last two are two standard metrics in image captioning. Here, Tanimoto was calculated using PubChem fingerprints of CDK after decoding DeepSMILES back to SMILES.

**Table 2:** Description of the training, validation, test set

Set	Category 1	Category 2	Category 3	Category 4	Total
Training	1,125,000	1,125,000	1,125,000	1,125,000	4,500,000
Validation	62,500	62,500	62,500	62,500	250,000
Test	62,500	62,500	62,500	62,500	250,000

### Tokenization

We counted all the characters of the DeepSMILES strings in our dataset. There were 76 unique characters. We treated each unique character as a token.

Tokens in our dataset: c, 6,), C, = , O, N, S, l, s, 5, B, r, n, [, H, + ,], %, 1, 0, /, \, R, F, #, 4, (, 9, -, @, L, 3, 8, 2, ', G, a, 7, Z,., P, t, Y, o, A, X, i, J, q, x, Q, m, b, d, E, w, I, V, z, e, M,,, D, K, p, v, h, y, u, g, k, T, W, U, f.

### Training

We used the same setting for each experiment to make a fair comparison. We employed a batch size of 256 images (224 × 224 pixels). An Adam [32] optimizer of an initial learning rate 5e−4 and token embedding dimension of 256 were used. The backbone and Transformer networks used cosine and step decays, respectively, regarding the learning rate scheduler. The loss function used the standard cross entropy (CE) loss. To prevent model overfitting during training, the dropout rate was set to 0.1. The model trained for 30 epochs on a server configured with NVIDIA Tesla V100-PCIE graphic cards.

### Experiments and results

We conducted experiments on our dataset for performance and analysis. Firstly, we evaluated the backbone performance of Swin Transformer, ResNet-50 and EfficientNet-B3. Then, we evaluate the CE loss and focal loss. Finally, we analyzed the influence of molecule category and DeepSMILES strings length.

In addition, because our model is trained on the generated training set, it may not achieve satisfactory results on real-world test sets which are derived from the literature. Therefore, we also performed experiments on a small real-world test set.

### Backbone comparison experiment

**Fig. 9:**
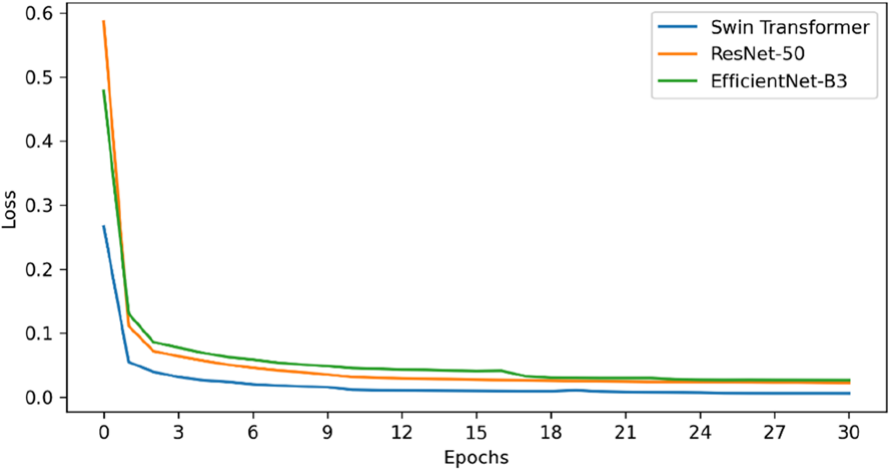
CE loss curve of training

**Fig. 10:**
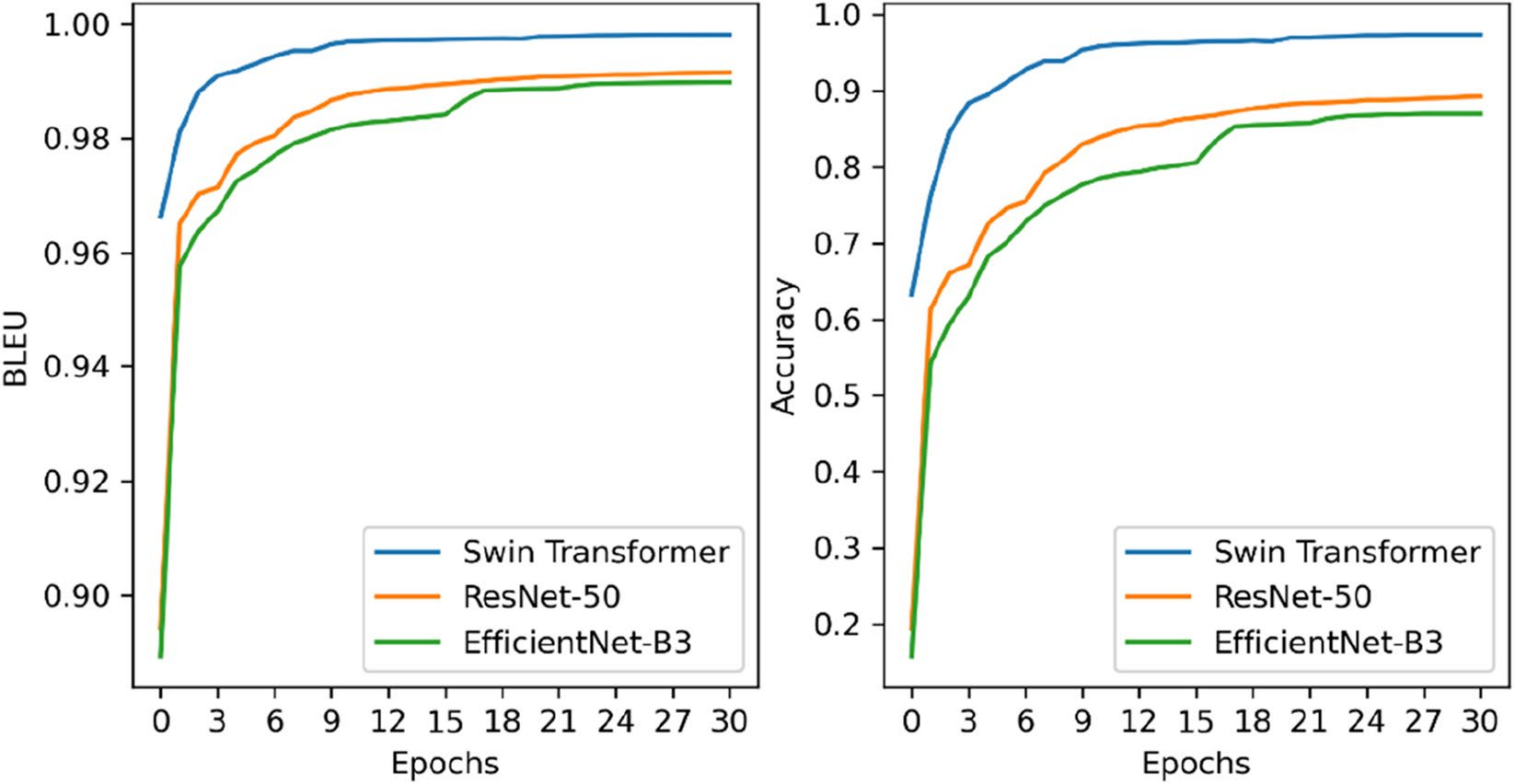
Validation curves (left: BLEU, right: Accuracy)

To evaluate the Swin Tranformer performance as our model’s backbone, we compared the Swin Transformer with two CNNs, ResNet-50 of Image2SMILES [16] and EfficientNet-B3 of DECIMER 1.0 [15]. CE loss curve of training are shown in Fig. 9. The loss value of our model (using Swin Transformer as the backbone) is smaller than those of ResNet-50 and EfficientNet-B3 in all cases, indicating that our model has a faster convergence. Validation curves (BLEU, accuracy) are shown in Fig. 10. Our model is still superior to the other two models in term of accuracy and BLEU score in all cases. The results demonstrate that our model has better data fitting ability. Finally, we made a comparison on the test set. As shown in Table 3, our model demonstrated the best performance based on all four metrics with a BLEU score of 99.46%, Tanimoto of 99.65%, ROUGE score of 99.64%, and accuracy of 97.36%. For accuracy, our model reached 97.36% and 8.19% and 10.66% higher than ResNet-50 and EfficientNet-B3, respectively. The accuracy metric requires that the predicted and actual DeepSMILES strings have the same character in each position. Hence, the metric is an essential requirement and can better reflect the performance of a model compared with the other three metrics.

**Table 3:** Backbone performance comparisons in the test set

Backbone	Accuracy	Tanimoto	BLEU	ROUGE
Swin Transformer(our)	**0.9736**	**0.9965**	**0.9946**	**0.9964**
ResNet-50	0.8917	0.9879	0.9862	0.9887
EfficientNet-B3	0.8670	0.9846	0.9837	0.9866

### Loss function comparison experiment

**Fig. 11:**
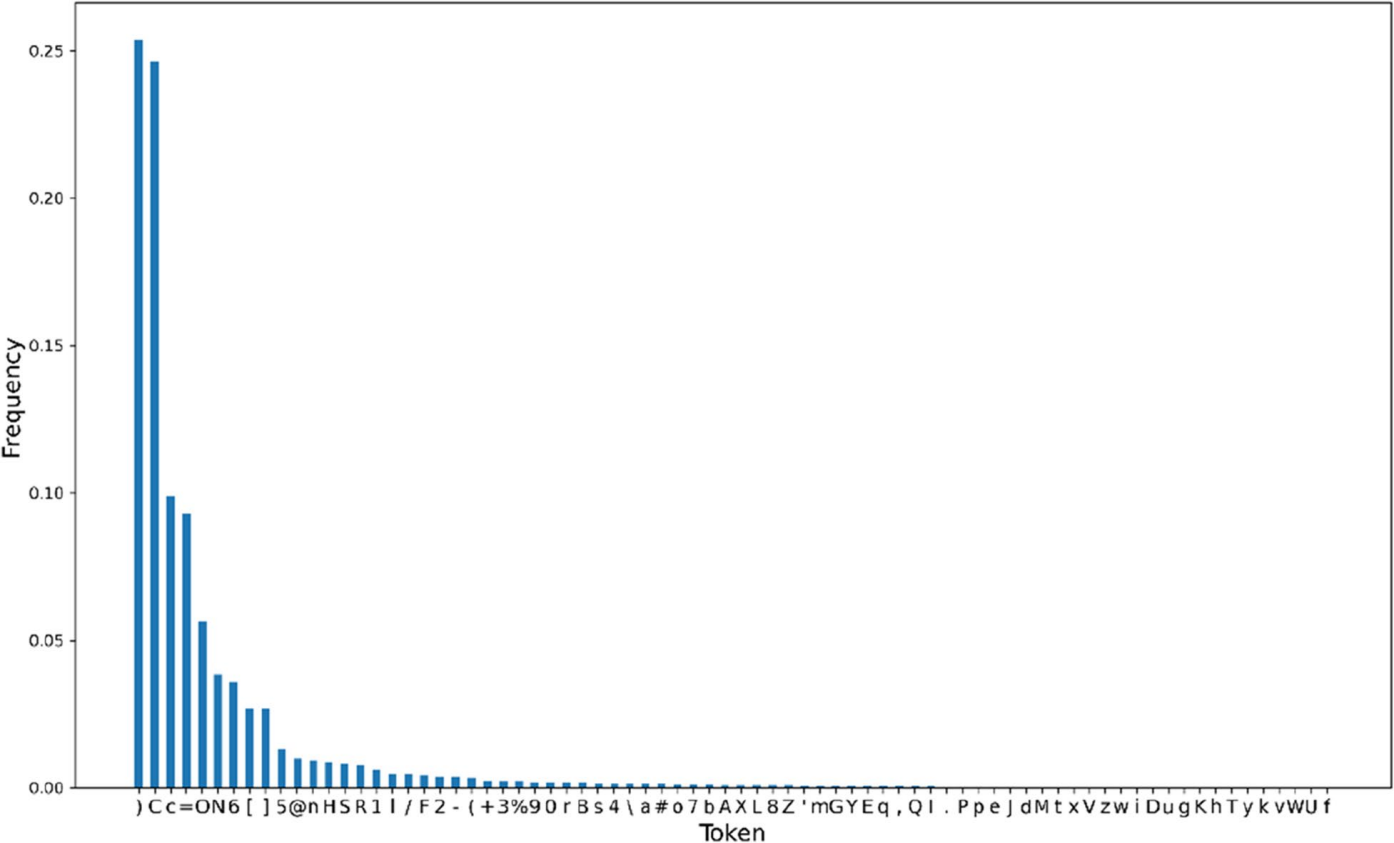
Frequency distribution histogram of tokens in our dataset

The frequency distribution of tokens affects the model’s performance when CE loss is used as the model’s loss function. On this basis, we counted the distribution of tokens in our dataset. The result is shown in Fig. 11, where the total number of tokens is 234706822. We found that the frequency distribution of tokens shows a long-tail distribution. A few tokens on the left, such as “),” “C,” “c,” and “ = ” formed the frequency header. Most tokens on the right formed the frequency tail. This indicates a significant imbalance of token classes in our dataset. Therefore, the model tends to predict a small number of token classes with high frequency during training. As a few token classes with high frequency contribute to the majority of the loss, even if the model ignores other token classes, the CE loss was not greatly affected.

To solve this problem, we used the focal loss [18], a common solution in object detection. Because focal loss is usually used for binary tasks, we converted our single-label classification task to multi-label classification task and rewrote the focal loss as multi-label focal loss (MFL). Given *n* classes, the model outputs one logit per class, *o*_*i*_. Each logit is independently activated by a sigmoid function *σ*(*o*_*i*_). The probability of each label, *p*_*i*_, is given by:

1 $$p_{i} = \left\{ {\begin{array}{*{20}l} {\sigma \left( {o_{i} } \right)} \hfill & {{\text{ if }}y_{i} = 1} \hfill \\ {1 - \sigma \left( {o_{i} } \right)} \hfill & {\text{ otherwise, }} \hfill \\ \end{array} } \right.$$

where *y*_*i*_ is the ground-truth label for class *i*. The average loss of binary loss per label, MFL, is obtained by:

2 $${\text{MFL}} = \frac{1}{n}\sum\limits_{i}^{n} { - \alpha_{i} \left( {1 - p_{i} } \right)^{\gamma } \log \left( {p_{i} } \right)} ,$$

where *α*_*i*_ is the weighting factor for class *i* and *γ* is a focusing parameter. In Table 4, we compare performance of MFL with CE on the  test set. It is evident that loss function using MFL outperforms CE on all four metrics. Because the model that uses MFL is our best model, we utilized SwinOCSR that uses MFL in the following experiments.

**Table 4:** Loss function performance comparisons

Loss	Accuracy	Tanimoto	BLEU	ROUGE
MFL	**0.9858**	**0.9977**	**0.9959**	**0.9978**
CE	0.9736	0.9965	0.9946	0.9964

### Influence of molecule category

To analyze the prediction performance of SwinOCSR on different molecule categories, the four data categories in the test set were evaluated separately based on accuracy. The result is shown in Table 5. Category 1 and 2 are lower than 3 and 4. demonstrating that SwinOCSR performs a little better on the data with substituents. We believe that the reason for this is that substituents will be explicitly reflected in the molecular diagram. Hence, the SwinOCSR is easier to extract information about substituents and identify them. Table 5 also shows that category 1 is lower than 2 and 3 is lower than 4, demonstrating that SwinOCSR performs a little better on the data with Aromatic rings. The explanation is that in molecular diagram, the Aromatic ring is presented as a circle distinct from other molecular diagram elements. In contrast, the Kekule ring is represented as lines similar to other elements of the molecule diagram. Therefore, the Aromatic rings that are distinct from other elements are easier to identify. There is not much difference in accuracy among four categories of data. This shows that SwinOCSR has good robustness with different categories of data.

**Table 5:** Performance of SwinOCSR on different categories of data

Category	Accuracy
1	0.9820
2	0.9846
3	0.9876
4	0.9889

### Influence of DeepSMILES string length

**Fig. 12:**
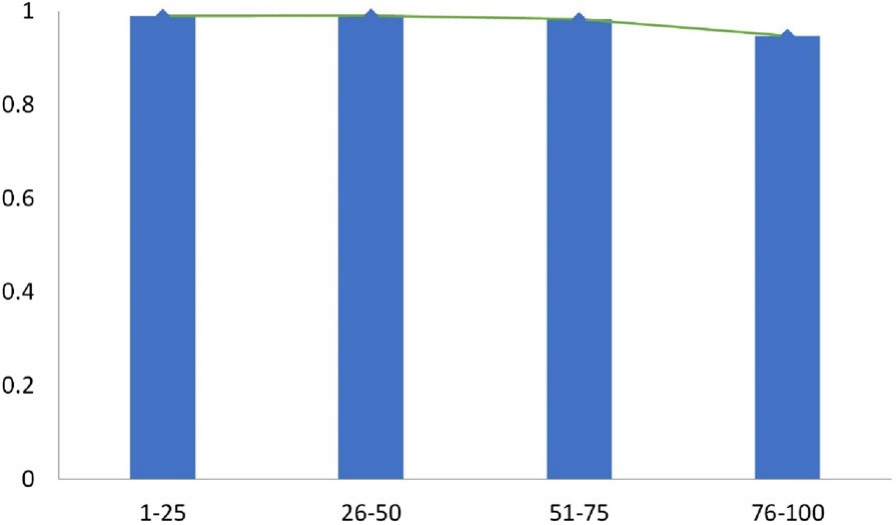
Performance of SwinOCSR with different DeepSMELES string lengths

To analyze the prediction performance of SwinOCSR on the DeepSMILES strings of different lengths, we divided the DeepSMILES strings of the test set into the following length ranges: [1, 25], [26, 50], [51, 75], [76, 100], and reported the accuracy within the ranges as the performance metric. A phenomenon to be expected is that the model performance declines as the DeepSMILES strings length increases, because the longer the DeepSMILES strings, the more times the model has to decode and the more likely errors will occur. The result is shown in Fig. 12. Moreover, SwinOCSR remains steady with [1, 75] and decreases slightly with [76, 100]. This indicates that SwinOCSR can adapt to changes in the length of the DeepSMILES strings. Even in the lowest range [76–100], SwinOCSR can still achieve an accuracy of 94.76%, indicating that it has a strong ability to recognize large molecules with long DeepSMILES strings. This shows that the backbone of SwinOCSR can extract richer chemical structure information from molecular graphs. Thus, more characters can be predicted during decoding.

### Performance on real data

To evaluate the prediction performance of SwinOCSR on real-world test sets, we have constructed a small real-world test set and conducted experiments on the test set. The small real-world test includes 100 images derived from the literature and their corresponding canonical SMILES strings which are manually labeled. The results are shown in Table 6. Our model achieved an accuracy of 25%, and the performance of our model on the real-world test set is unsatisfactory.

**Table 6:** Performance on the test set derived from the literature

Metric	Literature
Accuracy	0.2500
Tanimoto	0.5975
BLEU	0.7261
ROUGE	0.8058
Valid DeepSMILES	0.9800
Valid SMILES	0.9700

We also used CDK to generate images from the manual-labeled canonical SMILES strings of the small real-world test set, and constructed a new generated test set. We also conducted experiments on the generated test set, and the results are shown in Table 7. Our model achieved an accuracy of 94%, and the performance is good in term of all metrics.

**Table 7:** Performance on the test set generated by CDK

Metric	CDK
Accuracy	0.9400
Tanimoto	0.9906
BLEU	0.9905
ROUGE	0.9954
Valid DeepSMILES	1.0000
Valid SMILES	1.0000

We analyzed several molecule examples in the above experiments. Table 8 shows two examples that are correctly extracted in both the real-world test set from the literature and the test set generated by CDK, Table 9 demonstrates two examples that are incorrectly extracted in the real-world test set and are correctly extracted in the generated test set, and Table 10 shows one example that is incorrectly extracted in both the real-world test set and the generated test set.

**Table 8:** Two examples that are correctly extracted in both the test set from the literature and the test set generated by CDK

Items	Molecule 1	Molecule 2
The real-world image derived from the literature		
Manual-labeled SMILES	CNC1 = CC(= NC(= N1)C2 = CC = CC = C2)N3CCC(CC3)C(= O)NCC4 = CC = CC = C4C(F)(F)F	C2 = CC1 = CC(= CC = C1N = C2)CN4C3 = NC(= NC = C3N = N4)C5 = CN(CCO)N = C5
Predicted SMILES from the real-world image	CNC1 = CC(= NC(= N1)C2 = CC = CC = C2)N3CCC(CC3)C(= O)NCC4 = CC = CC = C4C(F)(F)F	C2 = CC1 = CC(= CC = C1N = C2)CN4C3 = NC(= NC = C3N = N4)C5 = CN(CCO)N = C5
Generated image from manual-labeled SMILES by CDK		
Predicted SMILES from the generated image	CNC1 = CC(= NC(= N1)C2 = CC = CC = C2)N3CCC(CC3)C(= O)NCC4 = CC = CC = C4C(F)(F)F	C2 = CC1 = CC(= CC = C1N = C2)CN4C3 = NC(= NC = C3N = N4)C5 = CN(CCO)N = C5

**Table 9:** Two examples that are incorrectly extracted in the test set from the literature and are correctly extracted in the test set generated by CDK

Items	Molecule 1	Molecule 2
The real-world image derived from the literature		
Manual-labeled SMILES	CC1 = CC = CC(= C1)N	C1 = C(N(C(= O)C(= C1[R3])[R4])[R8])[R]
Predicted SMILES from the real-world image	[CH3-]C1 = CC = CC(= C1)N.[I-]	CC(C)(C)C1 = CC(= CC(= O)N1[Rx])[R3]
Generated image from the above-mentioned predicted SMILES		
Generated image from manual-labeled SMILES by CDK		
Predicted SMILES from the generated image	CC1 = CC = CC(= C1)N	C1 = C(N(C(= O)C(= C1[R3])[R4])[R8])[R]

**Table 10:** One example that is incorrectly extracted in both the test set from the literature and the test set generated by CDK

Items	Molecule 1
The real-world image derived from the literature	
Manual-labeled SMILES	c1c(cc(c(c1[Y1])[X0])[Y2])c2c(cc([H][H][R0])cc2[Y4])[Y3]
Predicted SMILES from the real-world image	C1CC(CCC1C2CCC(CC2)[Y])c4cc(c(-c3cc(c(c(c3)[Y1])[Y1])[Y])c(c4)[Y])[Y]
Generated image from the above-mentioned predicted SMILES	
Generated image from manual-labeled SMILES by CDK	
Predicted SMILES from the generated image	c1c(cc(c(c1[Y1])[X0])[Y2])-c2c(cc(cc2[Y4])N[R0])[Y4]
Generated image from the above-mentioned predicted SMILES	

After analyzing, the unsatisfactory performance of our model on the real-world test set may be caused by the following three factors:

1. The images derived from the literature are vague, while the CDK-generated images are clearer.
2. The image derived from the literature is more complex while CDK-generated images are more regular.
3. Although a canonical SMILES string of a molecule ensures the unique SMILES representation of the molecule, there is no unique chemical structure representation for one specific molecule. There are a lot of image styles in generating images by different chemical programs. For example, some condensed structural formulas, such as NH, are expanded in CDK-generated images by default, and other condensed structural formulas, such as NO, NO2, CF3, CH3, etc., are unexpanded by default, so if the corresponding styles is changed, the image will be changed.

Of the above three factors, no unique chemical structure representation for one specific molecule is the most significant and more real-world chemical structures can alleviate the problem.

## Conclusion

In this study, we propose an end-to-end chemical structure image recognition approach, SwinOCSR, which can directly recognize the original chemical structure map without formulating manual features. Compared with existing approaches that use CNNs as the backbones, it achieved a high accuracy of 98.58%, superior performance, and fast convergence. It also performs well in recognizing long sequences, particularly in recognizing chemical structures containing substituents. Experimental results show that SwinOCSR can effectively extract the key features of chemical structures and capture the correspondence between chemical structure graphs and DeepSMILES.

However, despite the superior performance of our method on the generated data, the recognition performance in the literature is unsatisfactory. This can be attributed to some discrepancies existing between the chemical structures rendered by chemical software and those in the literature. For example, the real-world chemical structures in the literature have lower resolutions, various noises, and numerous complex patterns such as wavy lines, abbreviations, and superatoms. In fact, the performance of deep learning-based OCSRs depends on the model and the dataset. When our model achieved better performance on a generated dataset and demonstrated the model’s effectiveness, we believe that if there are enough real-world chemical structures to form a real-world training set and our model is trained on the training set, our model will also achieve better performance. Our model is a significant step toward the automatic extraction of real-world chemical structures. In the future, we will expand the data set to include as many low-resolution and complex chemical structure styles as possible. In addition, we hope to provide a software program that automates the extraction of chemical structures available in the literature. Finally, we hope that our work will open new possibilities for exploring end-to-end chemical structure recognition approaches.

## Data Availability

The dataset and source code supporting the conclusions of this article are available in the [SwinOCSR] repository, [unique persistent identifier and hyperlink to dataset in https://github.com/suanfaxiaohuo/SwinOCSR/tree/main].

## Appendix: 224 substituents

'[R]', '[R0]', '[R1]', '[R2]', '[R3]', '[R4]', '[R5]', '[R6]', '[R7]', '[R9]', '[R10]', '[R11]', '[R12]', '[R13]', '[R14]', '[R15]', '[R16]', '[R17]', '[R18]', '[R19]', '[R20]', '[R21]', '[R22]', '[R23]', '[R24]', '[R25]', '[R26]', '[R30]', '[R31]', '[R50]', '[R51]', '[R52]', '[R53]', '[R54]', "[R']", "[R2']", "[R4']", "[R7']", "[R8']", "[R9']", "[R10']", '[Ra]', '[Rb]', '[Rc]', '[Rd]', '[Rm]', '[Rn]', '[Rx]', '[R1a]', '[R1b]', '[R1c]', '[R1d]', '[(R1)s]', '[(R1)m]', '[R2a]', '[R2b]', '[R3a]', '[R3b]', '[R4b]', '[R4c]', '[R5a]', '[R8a]', '[R14a]', '[R4()x]', '[R()p]', '[Rc3]', '[Rc4]', '[Rc6]', '[Rc7]', '[Rc8]', '[(R1)a]', '[(R1)n]', '[(R2)b]', '[(R2)m]', '[(R2)n]', '[(R2)k]', '[(R3)c]', '[(R3)m]', '[(R3)n]', '[(R3)p]', '[(R3)q]', '[(R4)m]', '[(R4)q]', '[(R5)a]', '[(R5)n]', '[(R5)o]', '[(R5)p]', '[(R6)q]', '[(R5)s]', '[(R6)n]', '[(R7)d]', '[(R7)n]', '[(R11)r]', '[(R11)u]', '[(R12)r]', '[(R19)w]', '[(Rc)p]', '[(R21)p]', '[(R9)0–3]', '[(Ra)m]', '[(Ra)n]', '[(Rb)n]', '[(R2a)p]', '[(R2b)r]', '[(R4a)d]', '[(R4c)g]', '[(R4d)i]', '[(R)p]', '[(RD4)mD]', "[OR']", '[ORc]', '[(CR2)n]', '[CR1]', '[Z][R8]', '[Z1]', '[Z2]', '[Z3]', '[Z4]', '[Z5]', '[Z6]', '[Z7]', '[Z8]', '[Z9]', '[Z10]', '[(Z1)a]', '[(Z3)e]', '[(Z)n]', '[D1]', '[D2]', '[D3]', '[D4]', '[D5]', '[D6]', '[Y]', '[Y1]', '[Y2]', '[Y3]', '[Y4]', '[(Y)n]', '[Ar]', '[Ar1]', '[Ar2]', '[Ar3]', '[G]', '[G1]', '[G2]', '[G3]', '[G4]', '[(G)n]', '[X0]', '[X1]', '[X2]', '[X3]', '[X4]', '[X5]', '[X6]', '[Q]', '[Q1]', '[Q2]', '[L]', '[L1]', '[L2]', '[L3]', '[L4]', '[E]', '[E1]', '[E2]', '[A1]', '[A2]', '[A3]', '[A4]', '[A5]', '[A6]', '[A7]', '[A8]', '[(CH2)r]', '[(CH2)p]', '[(CH2)q]', '[(CH2)m]', '[(CH2)n]', '[(CH2)s]', '[(CH2)v]', '[(CH2)b]', '[(CH2)c]', '[(CH2)z]', '[(CH)n]', '[(C)t]', '[(C)m]', '[(C)n]', '[Hal]', '[M]', '[(L2)n]', '[J]', '[J1]', '[V]', '[()x]', '[B1]', '[B2]', '[B3]', '[U]', '[Het]', '[La]', '[Ea]', '[Eb]', '[Ec]', '[Lb2]', '[M1]', '[M2]', '[M3]', '[Xa]', '[Xb]', '[*]', '[**]', '[#]', '[XH]', '[(X)n]', '[(A1)e]', '[(A2)h]', '[Et]', '[Cy2]', '[a]', '[P1]', '[SOm]', '[F,Cl,Br,I]'.

## Abbreviations

OCSR: Optical chemical structure recognition
JEPG: Joint photographic experts group
PNG: Portable network graphics
GIF: Graphics interchange format
CNN: Convolutional neural network
RNN: Recurrent neural network
LSTM: Long short-term memory
GRU: Gated recurrent unit
NCI: National cancer institute
SDF: Simulation description format
MLP: Multilayer perceptron
CDK: Chemistry development kit
SMILES: Simplified molecular-input line-entry system
CE: Cross entropy
MFL: Multi-label focal loss
W-MSA: Window multi-head self attention
SW-MSA: Shift window multi-head self attention
